# Fat Quality Influences the Obesogenic Effect of High Fat Diets

**DOI:** 10.3390/nu7115480

**Published:** 2015-11-16

**Authors:** Raffaella Crescenzo, Francesca Bianco, Arianna Mazzoli, Antonia Giacco, Rosa Cancelliere, Giovanni di Fabio, Armando Zarrelli, Giovanna Liverini, Susanna Iossa

**Affiliations:** 1 Department of Biology, Federico II University, Via Cinthia, 80138 Naples, Italy; rcrescen@unina.it (R.C.); francibianco@yahoo.it (F.B.); arimazzoli@hotmail.it (A.M.); antonia.giacco@hotmail.it (A.G.); cancelliererosa@gmail.com (R.C.); liverini@unina.it (G.L.); 2 Department of Chemical Sciences, Federico II University, Via Cinthia, 80138 Naples, Italy; difabio@unina.it (G.D.F.); zarrelli@unina.it (A.Z.); 3 Inter-University Consortium SannioTech, Piazza San G. Moscati, 82030 Apollosa (BN), Italy

**Keywords:** high fat diet, unsaturated fatty acids, hepatic steatosis, lipid peroxidation

## Abstract

High fat and/or carbohydrate intake are associated with an elevated risk for obesity and chronic diseases such as diabetes and cardiovascular diseases. The harmful effects of a high fat diet could be different, depending on dietary fat quality. In fact, high fat diets rich in unsaturated fatty acids are considered less deleterious for human health than those rich in saturated fat. In our previous studies, we have shown that rats fed a high fat diet developed obesity and exhibited a decrease in oxidative capacity and an increase in oxidative stress in liver mitochondria. To investigate whether polyunsaturated fats could attenuate the above deleterious effects of high fat diets, energy balance and body composition were assessed after two weeks in rats fed isocaloric amounts of a high-fat diet (58.2% by energy) rich either in lard or safflower/linseed oil. Hepatic functionality, plasma parameters, and oxidative status were also measured. The results show that feeding on safflower/linseed oil diet attenuates the obesogenic effect of high fat diets and ameliorates the blood lipid profile. Conversely, hepatic steatosis and mitochondrial oxidative stress appear to be negatively affected by a diet rich in unsaturated fatty acids.

## 1. Introduction

Obesity is among the most relevant causes of morbidity and mortality in western countries and its incidence is steadily growing among adults as well as children [1]. The metabolic consequences of obesity are numerous, ranging from insulin resistance, low-grade inflammation, altered lipid profile, type 2 diabetes, cardiovascular disease, and hepatic steatosis [1,2,3]. Although obesity is considered a multi-factorial metabolic disturbance, prominent factors that determine the onset of obesity are the composition of the diet, the amount of ingested calories, and the level of physical activity [4].

In humans, obesity studies are difficult to be carried out taking into account all the above factors, mainly because they cannot be measured directly by the researcher, but rather they are referred by the subjects. Obviously, these factors exhibit a great degree of variability, so that obtained results are often contradictory. In this context, the use of animal models that very closely resemble the condition typical of human obesity can be very useful. A very good model for obesity studies is represented by laboratory rats [5,6], in which energy intake, diet composition, and the level of physical activity can be easily monitored and controlled. A gene-expression profiling study suggested that rats with diet-induced obesity represent an appropriate obesity model [7]. In addition, because of the standard housing conditions, laboratory rats exhibit sedentary behavior [8], similarly to what happens in humans.

In our previous studies, a high fat diet (HFD) was given to rats to elicit obesity and mimic the feeding style more common in western societies [9,10,11]. Even after short-term dietary treatment, rats developed obesity [10,11] and exhibited a decrease in oxidative capacity and an increase in oxidative stress in liver mitochondria [10]. However, it has pointed out that the harmful effects of a HFD could be different, depending on the type of fat included in the diet [12,13,14,15]. In fact, some authors have hypothesized that HFDs rich in unsaturated fatty acids are less deleterious for human health than those rich in saturated fat [12,13,14,15]. However, polyunsaturated fatty acids exhibit the highest sensitivity to reactive oxygen species (ROS)-induced damage, their sensitivity to oxidation exponentially increasing as a function of the number of double bonds per fatty acid molecule [16]. As a consequence, if antioxidant defense systems are unchanged, a higher degree of fatty acid unsaturation in cellular membranes may increase their sensitivity to lipid peroxidation and would also expose other molecules to lipoxidation-derived damage.

In the light of the above considerations, we think that it is important to assess whether changing the type of fat in the context of a high fat dietary regimen could differently affect respiratory capacity and oxidative stress of liver mitochondrial compartment. To this end, we compared the short term effects of two different HFDs, rich in lard (mainly monounsaturated and saturated fatty acids) or safflower/linseed oil (polyunsaturated fatty acids of omega-6 and omega-3 series).

## 2. Experimental Section

### 2.1. Animals and Treatments

Male Sprague-Dawley rats (Charles River, Italy) were caged singly in a temperature-controlled room (23 ± 1 °C) with a 12-h light/dark cycle (6.30–18.30). Treatment, housing, and euthanasia of the animals met the guidelines set by the Italian Health Ministry. All experimental procedures involving animals were approved by “Comitato Etico-Scientifico per la Sperimentazione Animale” of the University “Federico II” of Naples.

Rats were divided in two groups with the same mean body weight (250 ± 5 g) and were pair fed with 380 kJ metabolisable energy (ME)/day (corresponding to the spontaneous energy intake of the same rats, that was assessed before the start of the experiment) of a lard-based (L) or safflower-linseed oil based (S) diet for two weeks. The two HFDs contained 58.2% energy from fat, 21.1% energy from protein and 20.7% energy from carbohydrate and their composition is shown in Table 1. During the treatments, body weight, food, and water intake were monitored daily. Feces and urine were collected daily and the respective energy content assessed with a bomb calorimeter.

**Table 1:** Composition of experimental diets.

Component, g	Lard	Safflower-Linseed
Chow	57.0	57.0
Casein	13.1	13.1
Methionine	0.1	0.1
Choline	0.1	0.1
Vitamin mix	0.5	0.5
Mineral mix	1.8	1.8
Sunflower oil	1.4	1.4
Lard	26.0	-
Safflower oil	-	17.3
Linseed oil	-	8.7
Total weight	100.0	100.0
% ME		
Protein	21.1	21.1
Lipid	58.2	58.2
Carbohydrate	20.7	20.7
ME, kJ/100 g	1885	1885
Gross energy, kJ/100 g	2150	2150
Fatty acid composition, g/100 g fatty acid		
4:0–10:0	0.21	-
12:0	0.21	-
14:0	1.23	-
16:0	23.71	7.22
18:0	15.97	2.35
20:0	0.03	0.18
14:1n5	0.47	-
16:1n7	2.44	0.06
18:1n9	39.54	16.39
20:1n9	1.05	0.12
22:1n9	0.09	0.08
18:2n6	13.93	59.13
18:3n3	1.12	14.47
SFA%	41.4	9.8
MUFA%	43.6	16.6
PUFA%	15.0	73.6
UFA/SFA%	1.42	9.2

ME = metabolisable energy, SFA = saturated fatty acid, MUFA = monounsaturated fatty acid, PUFA = polyunsaturated fatty acid, UFA = unsaturated fatty acid.

At the end of the experimental period, the rats were euthanized by decapitation, blood and liver were collected, and the carcasses used for body composition determination.

### 2.2. Fuel Oxidation

The day before the sacrifice, 24-h VO_2_ and VCO_2_ of the rats were recorded with a four-chamber indirect open-circuit calorimeter (Panlab S.r.l., Cornella, Spain). Measurements were performed for the whole 24 h-period every 15 min for 3 min in each cage. Urine was collected for the whole (24-h) period and urinary nitrogen levels were measured by an enzymatic colorimetric method (FAR S.r.l., Settimo di Pescantina, Italy). Daily energy expenditure and substrate oxidation rates were calculated for the whole 24-h period from VO_2_, VCO_2_, and urinary nitrogen according to Even *et al.* [17].

### 2.3. Plasma Parameters

Blood samples were placed in EDTA-coated tubes and centrifuged at 1400 *g* for 15 min at 4 °C. Plasma concentrations of alanine aminotransferase (ALT), triglycerides, cholesterol and non-esterified fatty acids (NEFA) were measured by colorimetric enzymatic method using commercial kits (SGM Italia, Rome, Italy and Randox Laboratories ltd., Crumlin, UK). Lipid peroxidation was determined according to Fernandes *et al.* [18], by measuring thiobarbituric acid reactive substances (TBARS). Aliquots of plasma were added to 0.5 mL of ice-cold 40% trichloroacetic acid. Then, 2 mL of 0.67% of aqueous thiobarbituric acid containing 0.01% of 2,6-di-*tert*-butyl-*p*-cresol was added. The mixtures were heated at 90 °C for 15 min, then cooled in ice for 10 min, and centrifuged at 850× *g* for 10 min. The supernatant fractions were collected and lipid peroxidation was estimated spectrophotometrically at 530 nm. The amount of TBARS formed was calculated using a molar extinction coefficient of 1.56 × 10^5^ M^−1^ cm^−1^ for the malondialdehyde- thiobarbituric acid complex and expressed as nmol TBARS/mL.

### 2.4. Body Composition, Liver Composition, and Energy Balance

Guts were cleaned of undigested food and the carcasses were then autoclaved. After dilution in distilled water and subsequent homogenization of the carcasses with a Polytron homogenizer (Kinematica, Luzern, Switzerland), duplicate samples of the homogenized carcass were analyzed for energy content by bomb calorimeter. To take into account the energy content of liver, tissue samples were dried and the energy content was then measured with the bomb calorimeter. Total body and hepatic water content were determined by drying samples in an oven at 60 °C for 48 h. Total body and liver lipids were measured by the Folch extraction method [19]. The energy as lipid was calculated from body lipids by using the coefficient of 39.2 kJ/g, and was then subtracted from total body energy to obtain the energy as protein.

Liver triglycerides and cholesterol were measured by colorimetric enzymatic method using the above commercial kits.

Energy balance measurements were conducted by the comparative carcass technique over the experimental period, as detailed previously [20]. Briefly, gross energy density of the diets was measured by a bomb calorimeter and gross energy intake was calculated from daily food consumption. Then, ME intake was calculated by subtracting the energy measured in feces and urine from the gross energy intake. Gain of body energy and lipids was calculated as the difference between the final and initial content of body energy and lipids. Initial body energy and lipid content were obtained from a group of rats, sacrificed at the beginning of the dietary treatment. Metabolic efficiency was calculated as the percentage of body energy retained per ME intake and energy expenditure was determined as the difference between ME intake and energy gain.

### 2.5. Preparation of Liver Isolated Mitochondria and Measurement of Mitochondrial Oxidative Capacities and Proton Leak

Isolation of liver mitochondria and measurement of state 3 respiration were carried out as previously reported [9]. Briefly, liver tissue fragments were gently homogenized with a medium containing 220 mM mannitol, 70 mM sucrose, 20 mM 4-(2-hydroxyethyl)-1-piperazineethanesulfonic acid (HEPES), 1 mM ethylenediamine tetraacetic acid (EDTA), and 0.1% (*w*/*v*) fatty acid free bovine serum albumin (BSA), pH 7.4, in a Potter Elvehjem homogenizer set at 500 rpm (4 strokes/min). After withdrawing aliquots for further assays, the homogenate was then centrifuged at 1000× *g* for 10 min and the resulting supernatant was again centrifuged at 3000× *g* for 10 min. The mitochondrial pellet was washed twice and finally resuspended in a medium containing 80 mM KCl, 50 mM HEPES, 5 mM Tris-PO_4_, 1 mM ethylene glycol tetraacetic acid (EGTA), 0.1% (*w*/*v*) fatty acid-free BSA, pH 7.0. Oxygen consumption rate was measured polarographically with a Clark-type electrode (Yellow Springs Instruments, Yellow Springs, OH, USA) in a 3 mL-glass cell, at a temperature of 30 °C in a medium containing 80 mM KCl, 50 mM HEPES, 5 mM K_2_HPO_4_, 1 mM EGTA, 0.1% (*w*/*v*) fatty acid-free BSA, pH 7.0. All samples were allowed to oxidize their endogenous substrates for 3 min and then 10 mM succinate + 3.75 µM rotenone, 40 µM palmitoyl-carnitine + 2.5 mM malate or 10 mM glutamate + 2.5 mM malate were added as substrate. State 3 oxygen consumption was measured in the presence of 0.3 mM ADP. State 4 was obtained from oxygen consumption measurements at the end of state 3, when ADP becomes limiting and respiratory control ratio was calculated as state 3/state 4 ratio. Control experiments of enzymatic and electron microscopy characterization have shown that our isolation procedure (centrifugation at 3000× *g* for 10 min) results in a cellular fraction, which is essentially constituted by mitochondria.

If the activity of the respiratory chain is titrated with the inhibitor malonate, in the presence of oligomycin to prevent ATP synthesis, the resulting titration curve of membrane potential against respiration rate represents the kinetic response of the proton leak to changes in membrane potential. Mitochondrial oxygen consumption was measured polarographically with a Clark-type electrode, whereas mitochondrial membrane potential recordings were performed in parallel with safranin O using a dual-wavelength spectrophotometer (511–533 nm) as previously reported [9]. Titration of mitochondrial oxygen consumption and membrane potential were carried out at 30 °C by sequential additions of increasing malonate concentrations in a medium containing 80 mM LiCl, 50 mM HEPES, 5 mM TrisPO_4_, 1 mM EGTA pH 7.0, 10 mM succinate, 3.75 µM rotenone, 2 µg/mL oligomycin, 83.3 nmol/mg safranin O, 80 ng/mL nigericin, and 0.1% (*w*/*v*) fatty acid-free BSA. Titrations were carried out both in the absence and in the presence of fatty acid palmitate 17 µM. This concentration was selected to obtain a decrease in membrane potential, similar to that obtained in the transition from state 4 to state 3 condition (about 20 mV). Calibration curves made for each preparation were obtained from traces in which the extra-mitochondrial K^+^ level, [K^+^]_out_, was altered in the 0.1–20 mM range. The change in absorbance caused by the addition of 3 µM valinomycin was plotted against [K^+^]_out_. Then, [K^+^]_in_ was estimated by extrapolation of the line to the zero uptake point. The Nernst equation: ∆ψ = 61 mV log ([K^+^]_in_/[K^+^]_out_) was used to convert the absorbance readings into membrane potential values.

### 2.6. Fatty Acid Composition of Plasma, Hepatic Tissue and Hepatic Mitochondria

To obtain fatty acid composition of isolated mitochondria, liver tissue, and plasma, each sample was dissolved in dry methylene chloride and dried under a slight flow of nitrogen. The samples were dried for one hour over P_2_O_5_ and then treated for 30 min at 60 °C with a solution of boron trifluoride/methanol 10% (1.3 M, 2 mL) and 100 µL of dimethoxypropane. Finally, each solution was extracted twice with hexane and the organic phase was dried. The fatty acid methyl esters were re-dissolved in hexane, filtered on a millex and injected into a gas chromatograph. Gas chromatography analyses were obtained on an Shimadzu model GC2010 instrument equipped with a SP52–60 capillary column (Sigma-Aldrich, St Louis, MO, USA; 100 m × 0.25 inside diameter × 0.20 film thickness); flow rate 1.0 mL/min; injector temperature: 275 °C; splitting ratio: 1:20; detector temperature: 275 °C; carrier gas: helium for chromatography at a pressure of 1.8 psi; auxiliary gas: hydrogen for chromatography, under a pressure of 18 psi; air chromatography at a pressure of 22 psi; sensitivity of instrument: 4 to 16 times the minimum attenuation; amount of sample injected: 1.0 µL. Analyses were performed with the following temperature program: 175 °C for 10 min, 175–220 °C at 1.8 °C min^−1^, and 220 °C for 20 min. Fatty acid methyl esters were identified by comparing their retention times with those of 22 commercial fatty acid standards purchased from Supelco (Sigma-Aldrich Group, St. Louis, MO, USA), with the limit of quantitation of 14 ppb. Peroxidability index (PI) of fatty acids from plasma, liver, and hepatic mitochondria was calculated as reported by Feillet-Coudray *et al.* [21] to evaluate the changes in the susceptibility to oxidative damage induced by alterations of the fatty acid composition.

### 2.7. Hepatic Mitochondrial Lipid Peroxidation, Aconitase and Superoxide Dismutase (SOD) Specific Activity

Lipid peroxidation was determined in isolated mitochondria by using the same procedure used for plasma samples. The amount of TBARS formed was calculated using a molar extinction coefficient of 1.56 × 10^5^ M^−1^ cm^−1^ for the malondialdehyde-thiobarbituric acid complex and expressed as nmol TBARS/mg protein.

Active aconitase specific activity was measured spectrophotometrically by following the formation of NADPH (340 nm) at 25 °C in a mixture containing 0.2 mM NADP^+^, 5 mM sodium citrate, 0.6 mM MnCl_2_, 1 U/mL concentration of isocitric dehydrogenase, 50 mM Tris-HCl, pH 7.4 [22]. Aconitase inhibited by superoxide radical (O_2_^•−^) *in vivo* was reactivated so that total activity could be measured by incubating mitochondrial extracts in a medium containing 50 mM dithiothreitol, 0.2 mM Na_2_S, and 0.2 mM ferrous ammonium sulphate.

SOD specific activity was measured in a medium containing 0.1 mM EDTA, 2 mM KCN, 50 mM KH_2_PO_4_ pH 7.8, 20 mM cythocrome C, 0.1 mMxanthyne, and 0.01 units of xanthyne oxidase. Determinations were carried out spectrophotometrically (550 nm) at 25 °C by monitoring the decrease in the reduction rate of cythocrome C by superoxide radicals generated by the xanthine-xanthine oxidase system [23]. One unit of SOD activity was defined as the concentration of enzyme that inhibits cythocrome C reduction by 50% in the presence of xanthine + xanthine oxidase.

### 2.8. Western Blot Quantification of Uncoupling Protein 1 (UCP1) in Interscapular Brown Adipose Tissue (IBAT)

IBAT samples were homogenized in lysis buffer containing 20 mM Tris-HCl (pH 7.5), 150 mM NaCl, 2.7 mM KCl, 5% (*v*/*v*) glycerol, 1% (*v*/*v*) Triton X-100 and 50 µL/g tissue of protease inhibitor cocktail (all from Sigma-Aldrich, St. Louis, MO, USA) using a Potter homogenizer, shaken for 2 h at 4 °C, and centrifuged at 1,4000× *g* for 20 min at 4 °C. The supernatants were collected, aliquots were denatured in a buffer (60.0 mM Tris pH 6.8, 10% sucrose, 2% SDS, 4% β-mercaptoethanol) and loaded onto a 12% SDS-Polyacrylamide gel. After the run in electrode buffer (50 mM Tris, pH 8.3, 384 mM glycine, 0.1% SDS), the gels were transferred onto polyvinylidene difluoride membranes (Immobilon-P, Merck Millipore, Billerica, MA, USA) at 0.8 mA/cm^2^ for 90 min. The membranes were preblocked in blocking buffer (PBS; 5% milk powder; 0.5% Tween 20) for 1 h and then incubated overnight at 4 °C with a rabbit antibody for uncoupling protein 1 (UCP1) (Alpha Diagnostic International, San Antonio, TX, USA) at 1 µg/mL dilution in blocking buffer. The membranes were washed and then incubated for 1 h at room temperature with an anti-rabbit (horseradish peroxidase conjugated) secondary antibody (Promega Corporation, Madison, WI, USA). The membranes were finally washed, rinsed in distilled water, and incubated at room temperature with an enhanced chemiluminescent labeling, ECL (GE Healthcare, Amersham, UK). Data detection and acquisition have been carried out by using respectively Bio-Rad ChemiDoc Imaging and Quantity One Analysis Software (Bio-Rad Imaging and Software, Hercules, CA, USA). Quantifications of signals was carried out by Un-Scan-It Gel (Silk Scientific, Orem, UT, USA). To normalize the specific signal in each lane, actin was detected as above using a rabbit polyclonal antibody (Sigma-Aldrich) and an anti-rabbit (alkaline phosphatase) secondary antibody (Promega Corporation).

### 2.9. Statistical Analysis

Data are given as means with their standard errors. Statistical analyses were performed by two-tailed, unpaired, Student’s *t*-test. Probability values less than 0.05 were considered to indicate a significant difference. All analyses were performed using GraphPad Prism 6 (GraphPad Software, San Diego, CA, USA).

### 2.10 Materials

All chemicals used were of analytical grade and were purchased from Sigma (St. Louis, MO, USA).

## 3. Results

**Figure 1:**
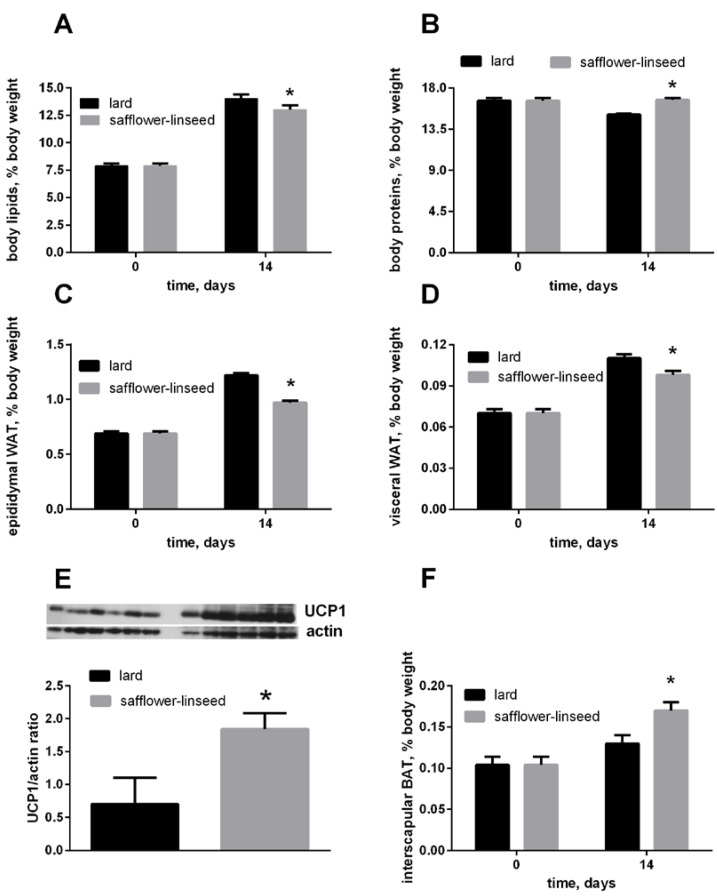
Body lipids (**A**) body proteins; (**B**) body epididymal WAT; (**C**) body visceral WAT (**D**) and body interscapular BAT (**F**) at day 0 and 14 of the experiment and UCP1 content in interscapular BAT (**E**) at day 14 of experiment in rats fed lard or safflower-linseed high fat diet. Values are reported as means with their standard errors. *n* = 8 different rats, except that for UCP1 western blots (*n* = 6). * *p* < 0.05 compared to lard. WAT = white adipose tissue, BAT = brown adipose tissue, UCP1 = uncoupling protein 1.

After two weeks of isocaloric high fat feeding, obesity development was evident both in L and S rats, since their percentage of body lipids about doubled compared to initial value, although the final value was significantly lower in S rats than in L rats (Figure 1A). In addition, the percent of epididymal and visceral white adipose tissue (WAT) increased during dietary treatment, reaching a final value that was significantly lower in S rats than in L rats (Figure 1C,D). The percent of IBAT was significantly higher in S rats than in L rats (Figure 1F), and its content of UCP1 was markedly increased in S rats compared to L rats (Figure 1E). Finally, the percent of body protein was maintained constant after two weeks of dietary treatment in S rats, while it significantly decreased in L rats (Figure 1B). As a consequence, S rats exhibited lower lipid gain and epididymal fat weight, while protein gain was significantly higher, compared to L rats (Table 2).

**Table 2:** Energy balance in rats fed lard or safflower-linseed high fat diet.

Energy Balance	Lard	Safflower-Linseed
Final BW, g	351 ± 4	345 ± 4
Liver weight, g/100 g BW	4.26 ± 0.21	4.45 ± 0.22
Epididymal fat weight, g/100 g BW	1.22 ± 0.05	0.97 ± 0.05 *
Visceral fat weight, g/100 g BW	0.111 ± 0.004	0.0980 ± 0.004 *
IBAT weight, g/100 g BW	0.45 ± 0.01	0.57 ± 0.01 *
BW gain, g	101 ± 2	94 ± 5
ME intake, kJ	5286 ± 158	5279 ± 143
Energy gain, kJ	1372 ± 67	1303 ± 87
Lipid gain, kJ	1125 ± 44	960 ± 66 *
Protein gain, kJ	244 ± 17	353 ± 16 *
Energy expenditure, kJ	3915 ± 52	4020 ± 113
Metabolic efficiency, %	26.0 ± 1.3	24.9 ± 1.3

Values are reported as means with their standard errors. *n* = 8 different rats. * *p* < 0.05 compared to lard. BW = body weight, ME = metabolisable energy; IBAT = interscapular brown adipose tissue.

**Figure 2:**
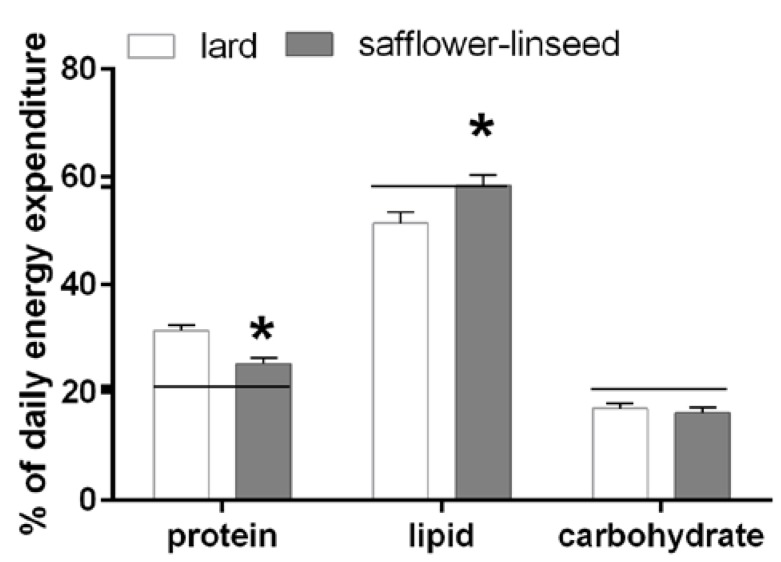
Percent contribution of lipids, proteins and carbohydrates to total daily energy expenditure (lard = 380 ± 15, safflower-linseed = 410 ± 25 kJ/day × kg^0.75^) in rats fed lard or safflower-linseed high fat diet. Values are reported as means with their standard errors. *n* = 8 different rats. * *p* < 0.05 compared to lard. Horizontal lines indicate the percent of each macronutrient in the diet (carbohydrate = 20.7%, protein = 21.1%, lipid = 58.2%).

Fuel oxidation was assessed the day before the sacrifice and it was found that S rats had reduced protein oxidation but higher lipid oxidation compared to L rats (Figure 2). In addition, fuel oxidation in S rats almost matched fuel composition of the diet, while in L rats fuel oxidation was lower than fuel composition of the diet for lipids and it was higher for proteins (Figure 2). Therefore, it appears clear that L rats exhibit an impaired metabolic flexibility that exacerbates obesity development.

**Figure 3:**
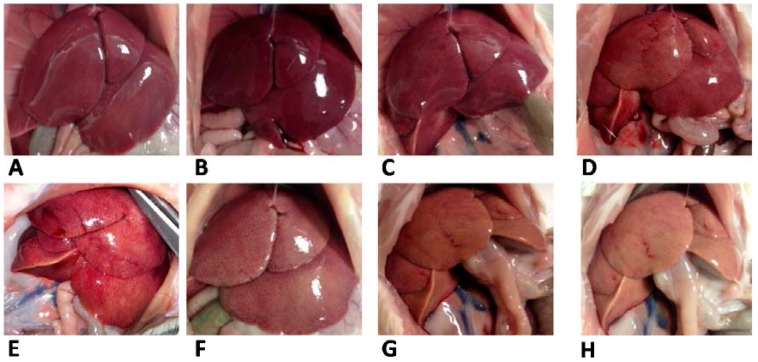
Liver appearance in rats fed lard (**A**–**D**) or safflower-linseed (**E**–**H**) high fat diet.

Plasma metabolic characterization evidenced lower cholesterol but higher lipid peroxidation and ALT activity in S rats compared to L rats (Table 3). At variance with plasma lipid profile, livers from S rats had higher lipids, triglycerides, and cholesterol, as well as higher lipid peroxidation, while water content was significantly lower, compared to L rats (Table 3). The different appearance of livers from L and S rats was evident from pictures taken at the time of sacrifice (Figure 3).

**Table 3:** Plasma parameters and liver composition in rats fed lard or safflower-linseed high fat diet.

	Lard	Safflower-Linseed
**Plasma parameters**		
Triglycerides, mg/dL	244 ± 15	244 ± 14
Cholesterol, mg/dL	143 ± 10	124 ± 11 *
Non esterified fatty acids, mmol/L	1.8 ± 0.4	1.8 ± 0.3
Lipid peroxidation, nmol TBARS/mL	13.2 ± 1.0	16.6 ± 0.8 *
ALT, U/L	7.2 ± 0.5	10.0 ± 0.6 *
**Hepatic composition**		
Water, mg/g	741 ± 20	670 ± 19 *
Lipids, mg/g	60.3 ± 5.3	86.9 ± 7.4 *
Triglycerides, mg/g	26.7 ± 1.8	38.5 ± 3.0 *
Cholesterol, mg/g	6.2 ± 0.6	10.3 ± 0.5 *
Lipid peroxidation, nmol TBARS/g	61.0 ± 2.0	103.4 ± 7.0 *

Values are reported as means with their standard errors. *n* = 8 different rats; * *p* < 0.05 compared to lard; ALT = alanine aminotransferase; TBARS = thiobarbituric acid reactive substances.

Both nicotinamide adenine dinucleotide (NAD), flavin adenine dinucleotide (FAD) and lipid-linked maximal (state 3) oxidative capacities were significantly higher in isolated liver mitochondria from S rats compared to L rats (Table 4).

**Table 4:** Mitochondrial respiratory capacities in rats fed lard or safflower-linseed high fat diet.

Respiratory Capacities	Lard	Safflower-Linseed
Glutamate + malate		
State 4	8.9 ± 0.9	10.3 ± 0.8
State 3	96.0 ± 7.1	120.9 ± 5.0 *
RCR	10.8	11.7
Palmitoyl carnitine + malate		
State 4	10.7 ± 0.8	12.6 ± 1.2
State 3	94.2 ± 6.2	118.0 ± 6.0 *
RCR	8.8	9.4
Succinate + rotenone		
State 4	26.6 ± 2.7	28.9 ± 2.1
State 3	153.8 ± 6.1	187.8 ± 6.0 *
RCR	5.8	6.5

Values are expressed as ngatoms of oxygen/(min × mg protein) and reported as means with their standard errors. *n* = 8 different rats; * *p* < 0.05 compared to lard; RCR = respiratory control ratio.

**Figure 4:**
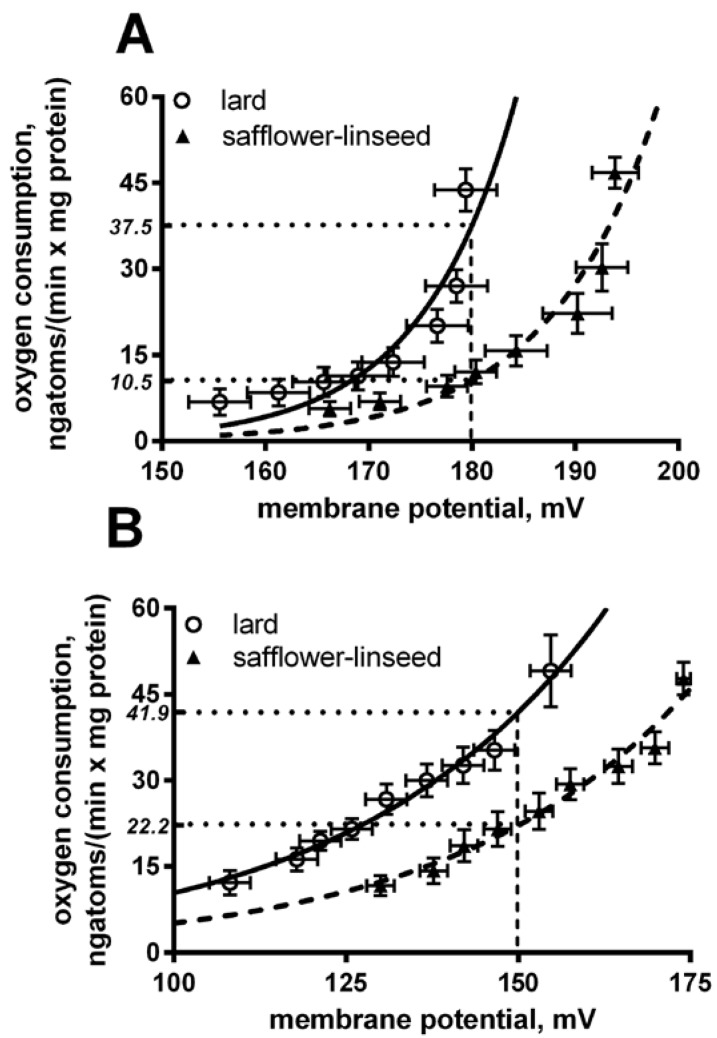
Basal (**A**) and fatty acid-induced (**B**) proton leak in rats fed lard or safflower-linseed high fat diet. Values are reported as means with their standard errors. *n* = 8 different rats. Non-linear regression analysis show that curves for rats fed lard were significantly different than those of rats fed safflower-linseed (*p* < 0.05). In addition, at the same membrane potential (180 mV for basal leak) the oxygen used to balance proton leak is significantly (*p* < 0.05) higher in lard compared to safflower-linseed (lard = 37.5 ± 2.1; safflower-linseed = 10.5 ± 0.9 ngatoms oxygen/min × mg protein). Similarly, at the same membrane potential of 150 mV for fatty acid-induced proton leak, the oxygen used to balance proton leak is significantly (*p* < 0.05) higher in lard compared to safflower-linseed (lard = 41.9 ± 2.2; safflower-linseed = 22.2 ± 1.1 ngatoms oxygen/min × mg protein).

Liver mitochondria from S rats displayed a significant decrease in basal and fatty acid-induced proton leak compared to L rats (Figure 4). In fact, at the same membrane potential (180 mV for basal leak) the oxygen used to balance proton leak is significantly (*p* < 0.05) higher in lard compared to safflower-linseed (lard = 37.5 ± 2.1; safflower-linseed = 10.5 ± 0.9 ngatoms oxygen/min × mg protein). Similarly, at the same membrane potential of 150 mV for a fatty acid-induced proton leak, the oxygen used to balance the proton leak is significantly (*p* < 0.05) higher in lard compared to safflower-linseed (lard = 41.9 ± 2.2; safflower-linseed = 22.2 ± 1.1 ngatoms oxygen/min × mg protein).

**Figure 5:**
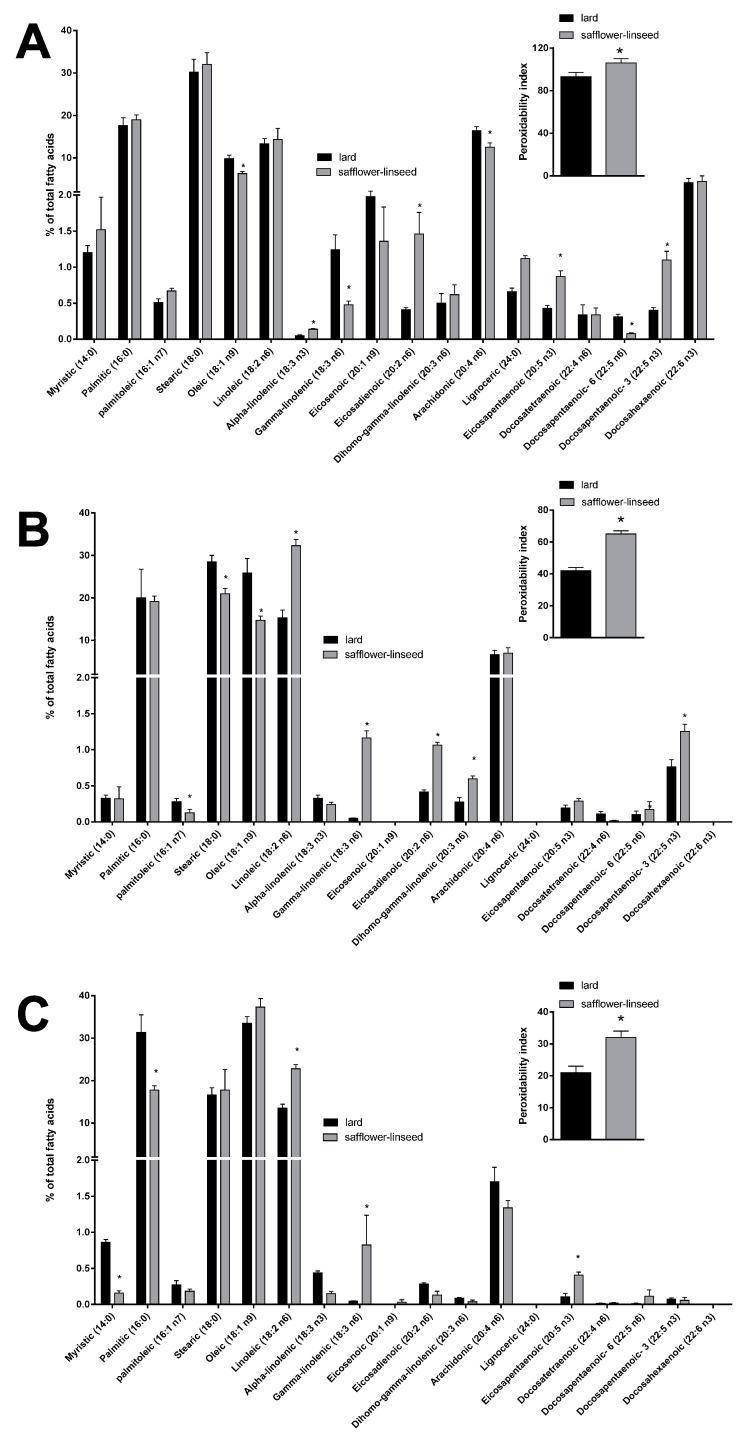
Percentage molar distribution of fatty acids and peroxidative index (inset) in hepatic mitochondria (**A**), hepatic tissue (**B**), and plasma (**C**) of rats fed lard or safflower-linseed high fat diet. Values are reported as means with their standard errors. *n* = 8 different rats. * *p* < 0.05 compared to lard.

Mitochondrial fatty acid composition was found to be quite similar between the two groups of rats (Figure 5A). However, some differences were evident in the content of specific fatty acids, such as the monounsaturated fatty acid oleic acid and the omega 6 fatty acids gamma linolenic, arachidonic, and docosapentaenoic, that were found to be significantly decreased, while the omega 3 fatty acids alpha linolenic, eicosapentaenoic, and docosapantaenoic were found to be significantly higher, in S rats compared to L rats (Figure 5A). As for fatty acid composition of liver tissue, a significant decrease was found in palmitoleic, stearic, and oleic acid content, while there was a significant increase in the omega 6 fatty acids linoleic, gamma-linolenic, eicosadienoic, and dihomo-gamma linolenic and in the omega 3 fatty acid docosapentaenoic (Figure 5B), in S rats compared to L rats. Fatty acid composition of plasma revealed a significant decrease in saturated fatty acids myristic and palmitic, together with a significant increase in omega 6 fatty acid linoleic and gamma-linolenic and omega 3 fatty acid eicosapentaenoic, in S rats compared to L rats (Figure 5C). Significantly higher values of PI were found in mitochondria, liver, and plasma samples from S rats compared to L rats (Figure 5).

Mitochondrial oxidative status was assessed taking into account oxidative damage to lipids through the determination of lipid peroxidation, oxidative damage to proteins through the measurement of the marker enzyme aconitase and antioxidant defense through the determination of SOD activity. Lipid peroxidation was significantly higher in mitochondria from S rats compared to L rats, while aconitase damage and SOD activity were the same between the two groups of rats (Table 5).

**Table 5:** Oxidative status in liver mitochondria from rats fed lard or safflower-linseed high fat diet.

Oxidative Status	Lard	Safflower-Linseed
Active aconitase, mU/mg protein	3.9 ± 0.2	3.9 ± 0.1
Total aconitase, mU/mg protein	10.9 ± 0.4	11.2 ± 0.3
Active/total aconitase ratio	0.36 ± 0.02	0.35 ± 0.02
Lipid peroxidation, nmol TBARS/mg protein	0.25 ± 0.01	0.35 ± 0.01 *
SOD specific activity, U/mg protein	81.7 ± 6.0	87.1 ± 7.1

Values are reported as means with their standard errors. *n* = 8 different rats. * *p* < 0.05 compared to lard. TBARS = thiobarbituric acid reactive substances, SOD = superoxide dismutase.

## 4. Discussion

Obesity is a condition of imbalance between intake and oxidation, in terms of energy and/or fuels [24]. In fact, there is general agreement that obesity arises when energy intake exceeds energy expenditure, but the degree of obesity development also depends on the ability to change the mix of oxidized substrates in response to changes in dietary composition [24]. Metabolic flexibility is defined by the capacity of the body or cells to match fuel oxidation to fuel availability and is typically assessed by the macronutrient oxidation adaptation in response to isoenergetic changes in diet composition [24]. In the context of the effect of isoenergetic changes in diet composition, an impaired metabolic flexibility may induce macronutrient imbalance. Under body weight stability, the food quotient (respiratory quotient, RQ, of the diet) matches 24-h RQ, and therefore macronutrient oxidation rates [25]. When the macronutrient composition of the diet is modified, fuel oxidation must be adjusted to achieve a new equilibrium [25]. The rate at which this adjustment is achieved is faster in the transition from a low-carbohydrate to high-carbohydrate diet; however, in response to an increase in dietary fat content, fat oxidation can take more than one week to match fat intake [24,25]. Here we show that this adjustment is also dependent on the type of fat contained in the HFD. In fact, when the HFD is rich in unsaturated fatty acids (S rats) the balance between the mixture of ingested substrates and the mix of oxidized fuels is achieved already after two weeks, while with HFD rich in saturated and monounsaturated fat (L rats) this period is not enough to achieve balance. As a consequence, in L rats the lipid oxidation rate is lower than the lipid intake. This imbalance has two metabolic consequences: the first one is that in L rats the extra lipid intake that is not oxidized is deposited as body lipids, thus increasing the obesogenic effect of the lard diet, while the second one is that the lower lipid oxidation in L rats is balanced in terms of calories by increasing the oxidation of proteins, so that body protein mass is decreased in these rats. The two metabolic consequences are very harmful for health maintenance, since increased lipid mass leads to obesity and decreased protein mass leads to decreased metabolic activity, thus exacerbating the obesogenic effect of the HFD. Both saturated and monounsaturated fatty acids, abundant in our lard-based HFD, may contribute to these metabolic alterations, since it has been shown that oleic acid also contributes to obesity outcomes [13]. The above metabolic disturbances are partly avoided with the HFD rich in unsaturated fat, since the balance between ingested and oxidized fat not only attenuates obesity development but also allows for the maintenance of body proteins. In searching for the cellular site that determines the lower obesogenic potential of the HFD rich in unsaturated fat, we looked at brown adipose tissue (BAT), a well-known thermogenic organ, that allows rodents to thrive in the cold and that is believed to contribute to diet-induced thermogenesis [26]. In recent years, the interest in the biology of BAT has again raised, due to the observation that this tissue can also be induced in humans [27]. In this study we have found that S rats display higher amount of BAT, as well as a significantly higher content of UCP-1, the mitochondrial protein that allows the dissipation of energy in the form of heat rather than ATP during oxidative phosphorylation [28]. We can thus speculate that a high presence of unsaturated fatty acids in the diet could represent a proliferative stimulus for BAT growth, as well as for UCP-1 upregulation. In fact, it has been shown that polyunsaturated fatty acids are effective in increasing UCP content in BAT [29]. In addition, polyunsaturated fatty acids are stronger ligands of ligSands of peroxisome proliferator activated receptor (PPAR) alpha and PPAR delta than monounsaturated or saturated fatty acids [30], and PPAR alpha [31] and PPAR delta [32] orchestrate the UCP1 gene induction. As a consequence, the thermogenic potential of the organ is significantly increased, thus allowing a higher lipid oxidation rate that contributes to reaching lipid balance. When this mechanism fails, such as when the diet is rich in saturated fat, the obesogenic effect of HFD increases.

The beneficial effects seen at the level of energy balance with the HFD rich in unsaturated fat are however counterbalanced by a worsening of plasma and liver metabolic profile. In fact, although plasma cholesterol was lowered by an unsaturated fat-rich diet, the systemic oxidative stress was significantly higher. A major factor contributing to the increased systemic oxidative stress is the significant increase in polyunsaturated fatty acid content and the PI of plasma samples from S rats, since polyunsaturated fatty acids are more prone to be oxidized by ROS. Similarly, in liver there is a significant increase in polyunsaturated fatty acid content and the PI in S rats compared to L rats, associated with a higher degree of steatosis, oxidative stress, and necrosis. Thus it appears that the liver is damaged by the flux of dietary polyunsaturated fatty acids. The increased hepatic steatosis in S rats could be due to the lower hepatic content of monounsaturated fatty acid oleic acid by us found in this rats. In fact, this decrease could give rise to a reduced very low density lipoprotein (VLDL) secretion, since it has been shown that oleic acid is important for this process [33]. Another factor that has been associated with increased lipid storage is *n*6/*n*3 ratio [34,35], that for hepatic lipids is significantly higher in S than in L rats (S = 24 ± 1, L = 18 ± 1, *p* < 0.05). Therefore, diets containing elevated amounts of polyunsaturated fats could represent a predisposing factor for the development of liver steatosis and associated liver disease.

Liver is a primary metabolic site and plays a central role in the regulation of fuel handling, so that an impairment of its metabolic activity would have a deep impact on whole body homeostasis. Due to their high metabolic activity, liver cells are rich in mitochondria, and changes in mitochondrial function would exert a greater effect of liver metabolic activity. The composition of the mitochondrial membranes is among the factors that could influence mitochondrial function [36,37]. For this reason, we assessed whether changes in the fatty acid composition of dietary lipids could influence mitochondrial composition and function. This issue is of relevance, since unsaturated fatty acids are more prone to oxidative damage and mitochondria are the primary cellular site where ROS are produced. As for mitochondrial composition, we found an altered phospholipid composition with a significant increase in the PI, as well as a greater degree of oxidative damage to the lipid component of the mitochondria, with no difference in the oxidative damage to proteins. Therefore, it appears that the altered composition of the mitochondrial membrane contributes to the increased lipid oxidative damage of these organelles. The differences found by us in fatty acid composition of mitochondrial membranes can be explained by taking into account that after the *de novo* synthesis of phospholipids, many of them undergo acyl chain remodeling, known as Lands’ cycle [37]. Therefore, fatty acid composition of the diet can affect the acyl chain remodeling of phospholipids, given that the liver mitochondrial phospholipids contain components of half-lives 1–6 and 10 days [38]. In addition, it has been shown that the fatty acid composition of phospholipids is not completely related to the presence of a given fatty acid in the diet, but there is a selection for individual fatty acids, since, for example, the incorporation of 18:2 was found to be similar, despite diets varying ~4-fold in this essential fatty acid [39]. Finally, *n*-3 PUFAs may competitively inhibit n-6 fatty acid metabolism by delta-6 desaturase [40] and this could explain our findings.

In mitochondria, ROS production arises from the reduced components of the respiratory chain, from which electrons are released and react with molecular oxygen, giving rise to the superoxide radical. It follows that, the greater the degree of reduction of respiratory chain complexes, the greater the production of ROS. In addition, it is well established that there is a strong positive correlation between membrane potential and ROS production [41,42]. It has been reported that, at high membrane potentials, even a small increase in membrane potential gave rise to a large stimulation of ROS production [41,42]. Similarly, only a small decrease in membrane potential was able to inhibit ROS production [41,42], so ‘mild uncoupling’, *i.e.*, a small decrease in membrane potential, was suggested to have a natural antioxidant effect [41,42]. In this context, the two proton conductance pathways existing in mitochondria, *i.e.*, basal proton leak and fatty acid induced proton leak, play a role in the control of ROS production. We assessed both components of proton leak, that were found significantly lower, while state 3 was significantly higher in liver mitochondria from S rats. The increased respiratory chain activity coupled with a decreased inner membrane permeability may give rise to increased ROS production, although a direct measurement of this parameter was not carried out. The decrease in proton leak could be related to the decreased arachidonic acid content found in mitochondrial phospholipids from S rats, since it has been reported that arachidonic acid content is positively correlated with proton flux [43,44] and Stillwell *et al.* [45] have shown that mitochondria from old rats contain more arachidonic acid and have lower mitochondrial coupling. An increased ROS formation by NADPH oxidase may also occur, since this class of enzymes has been implicated in the condition of oxidative stress associated with obesity [46,47]. However, it has been reported that MitoQ (mitochondrial ROS scavenger) was able to attenuate body weight gain and glucose intolerance provoked by high fat diet, while Apocynin (NADPH oxidase inhibitor) and Allopurinol (xanthine oxidase inhibitor) showed limited effects, thus suggesting secondary roles of xanthine oxidase or NADPH oxidase-dependent ROS production in the onset of oxidative stress-dependent obesity, glucose intolerance, and hepatic steatosis process [48].

The proton leak pathways are important determinants of the degree of coupling of the mitochondrial oxidative phosphorylation and of the quantity of substrates that these organelles burn when they produce the ATP needed to support the cellular ATP turnover. In fact, ATP hydrolysis dictates the rate of ATP synthesis, but the degree of coupling of mitochondria in turn dictates how much fuels are needed to obtain a certain amount of ATP. If mitochondria are more coupled, less energy coming from fuels is lost as heat during oxidative phosphorylation and less fuels are needed to obtain ATP, so that less fuels are oxidized and more lipids are deposited as ectopic fat. Therefore, the lower proton leak and the following greater coupling of liver mitochondria from S rats could explain the greater degree of steatosis found in these rats. In addition, since oxidative stress and mitochondrial dysfunction contribute to liver injury and inflammation [49], it can be speculated that on longer periods the hepatic steatosis of S rats could evolve into non-alcoholic steatohepatitis.

## 5. Conclusions

In conclusion, our present results suggest that not only the amount but also the type of dietary fat is a primary obesogenic factor. Dietary unsaturated fatty acids limit the development of fat mass and preserve lean mass, but liver appears to be negatively affected. In fact, hepatic steatosis induced by HFD is worsened by unsaturated fatty acids, and mitochondrial oxidative stress develops together with increased efficiency of oxidative phosphorylation. Therefore nutritional strategies aiming at reducing the obesogenic effect of diet should take precautions in advising high intake of unsaturated fatty acids.
